# Type IV collagen-derived angiogenesis inhibitor: canstatin low expressing in brain-invasive meningiomas using liquid chromatography–mass spectrometry (LC-MS/MS)

**DOI:** 10.1007/s11060-023-04256-z

**Published:** 2023-02-22

**Authors:** Jian Pei, Pei Li, Yun H. Gao, Bao G. Tian, Da Y. Wang, Yu Zheng, Li Y. Liu, Zhi Y. Zhang, Si S. Huang, Min Wen, Xiang Xu, Lei Xia

**Affiliations:** 1 Department of Neurosurgery, Tangshan Gongren Hospital, Tangshan, 063000 People’s Republic of China; 2 Department of Neurology, Tangshan Gongren Hospital, Tangshan, 063000 People’s Republic of China; 3 Department of pathology, Tangshan Gongren Hospital, Tangshan, 063000 People’s Republic of China; 4 Department of pathology, The First Affiliated Hospital of Wenzhou Medical University, Wenzhou, 325000 People’s Republic of China; 5 Department of Neurosurgery, School of Medicine, Guangzhou First People’s Hospital, South China University of Technology, Guangzhou, 510000 People’s Republic of China; 6 Zhejiang Provincial Key Laboratory of Aging and Neurological Disorder Research, The First Affiliated Hospital of Wenzhou Medical University, Wenzhou, 325000 People’s Republic of China

**Keywords:** Meningioma, Brain invasion, Angiogenesis inhibitor, Canstatin, LC-MS/MS

## Abstract

**Purpose:**

Brain invasion in meningiomas is considered an indicator of more aggressive behavior and worse prognosis. But the precise definition and the prognostic role of brain invasion remains unsolved duo to lacking a standardized workflow of surgical sampling and the histopathological detection. Searching for molecular biomarker expression correlating with brain invasion, could contribute to establish a molecular pathological diagnosis without problems of subjective interobserver variation and deeply understand the mechanism of brain invasion and develop innovative therapeutic strategies.

**Methods:**

We utilized liquid chromatography tandem mass spectrometry to quantify protein abundances between non-invasive meningiomas (n = 21) and brain-invasive meningiomas (n = 21) spanning World Health Organization grades I and III. After proteomic discrepancies were analyzed, the 14 most up-regulated or down-regulated proteins were recorded. Immunohistochemical staining for glial fibrillary acidic protein and most likely brain invasion-related proteins was performed in both groups.

**Results:**

A total of 6498 unique proteins were identified in non-invasive and brain-invasive meningiomas. Canstatin expression in the non-invasive group was 2.1-fold that of the brain-invasive group. The immunohistochemical staining showed canstatin expressed in both groups, and the non-invasive group showed stronger staining for canstatin in the tumor mass (*p* = 0.0132) than the brain-invasive group, which showed moderate intensity.

**Conclusion:**

This study demonstrated the low expression of canstatin in meningiomas with brain invasion, a finding that provide a basis for understanding the mechanism of brain invasion of meningiomas and may contribute to establish molecular pathological diagnosis and identify novel therapeutic targets for personalized care.

**Supplementary Information:**

The online version contains supplementary material available at 10.1007/s11060-023-04256-z.

## Introduction

Most of meningiomas are slow-growing benign tumor and do not disrupt the surrounding brain tissue. Complete microsurgical excision is sufficient for curing the majority of the patients and contribute to good prognosis. But a subset of meningiomas have brain-invasive behavior [1] and worse prognosis [2]. Even after Simpson I resections, brain-invasive meningiomas have higher incidence of recurrence. As the clinical importance of brain invasion (BI) of meningiomas increased, it is believed being independently correlated with recurrence and became a stand-alone criterion for grade II meningiomas in the 2016 WHO classification of central nervous system (CNS) tumors [1–4]. But its prognostic impact remains controversial, based on contradictory results from various studies [5–8]. Inconstant assessment of BI from non-standardized tumor sampling and no clear-cut histopathological detection criteria may be a major key point causing the debate [2, 6, 9, 10]. In order to avoid the problem of non-standardized process in histopathological diagnosis, it is promising to establish molecular diagnosis by focusing on molecular mechanism and searching for molecular biomarker that could correlate with brain invasion.

BI in meningiomas involves molecular alterations at various cellular components and in signal transmission pathways which is related degradation of extracellular matrix/basement membrane (ECM/BM), and tumor cells migration and adhesion. By proteomic analysis, such as liquid chromatography–mass spectrometry (LC-MS/MS), these molecular alterations can be identified as biomarkers of BI of meningioma and therapeutic targets. It could contribute to establish molecular diagnosis to avoid the problematic sampling of histopathological investigation.

So, this study aimed to find difference of protein molecular expression between non-invasive and brain-invasive meningiomas using LC-MS/MS-based proteomics and try to contribute to molecular pathological diagnosis of BI and targeting therapy.

## Materials and methods

We identified data from patients with histopathological diagnosis of meningioma obtained from the Department of Neurosurgery of the TangShan GongRen Hospital between 2005 and 2021. Among them, we selected 21 cases with meningiomas grade III as invasive meningioma group that are characterized by aggressive behavior presenting as loss of cerebrospinal fluid (CSF) cleft, peritumoral edema in preoperative MRI, the remarkable adhesion and invasion of surrounding brain tissues in intraoperative findings, pathologically reported meningioma cell invading into adjacent brain, while the other 21 cases with convex meningiomas grade I as non-invasive group that are characterized by well-circumscribed CSF cleft in preoperative MRI, and complete arachnoidal interface in intraoperative findings, pathologically reported no BI when sampling the brain-tumor interface of adhesion areas. All samples were stored in liquid nitrogen immediately after removal.

### Immunoprecipitation

The samples used for the LC-MS/MS were all tumor tissue and did not contain any peripheral areas with a predominant proportion of normal brain tissue. Tissue mixtures were lysed in five volumes of lysis buffer (25 mM Tris-HCl, 150 mM NaCl, 3 mM MgCl2, 5% glycerol, 0.5% Nonidet P-40, 1 mM dithiothreitol, 1% protein inhibitor [PI], pH 7.4) for 2 h with rotation at 4 °C. Tissue lysates were cleared by centrifugation at 21,000 g for 30 min, followed by measurement of the supernatant using a 2-D quantitative kit. Equal amounts of protein were incubated with the ANTI-FLAG M2 affinity gel (Sigma) at 4 °C overnight, washed thrice with a wash buffer (25 mM Tris-HCl, 150 mM NaCl, 3 mM MgCl2, 0.2 mM EDTA, 0.1% Tween-20, 5% glycerol, 1% PI, pH 7.4) for 10 min, and proteins were eluted with 20 µL elution buffer containing 400 µg/mL 3×FLAG peptides (ChinaPeptides Co., Ltd.).

Immunoprecipitated proteins were separated by 12.5% SDS-PAGE gel and visualized using a Silver Staining Kit (Beyotime). The gels were de-stained and dehydrated, and the proteins were digested using sequencing-grade trypsin (Promega). The peptides were extracted from gel pieces with 0.1% formic acid (FA) and 50% acetonitrile and dried in a vacuum centrifuge (Thermo Fisher Scientific).

The peptides were dissolved in 10 µL of 0.2% FA and separated using an online Nano-LC system (Microtech Scientific) equipped with a C18 reverse-phase column. The column was eluted using a linear gradient of 5–30% acetonitrile in 0.2% FA at a rate of 500 nL/min for 100 min. Mass spectra were acquired using an LTQ-Orbitrap mass spectrometer (Thermo Fisher) equipped with a nano-ES ion source (Proxeon Biosystems). Full scan spectra (from m/z 300–1600) were acquired in an Orbitrap analyzer with a resolution of 60,000 at 400 m/z after the accumulation of 1,000,000 ions. The five most intense ions per scan were selected for collision-induced dissociation fragmentation in the linear ion trap after the accumulation of 3000 ions. We set the maximal filling times to 500 ms for the full scans and 150 ms for the LC-MS/MS scans. The dynamic exclusion list was restricted to a maximum of 500 entries, with a maximum retention period of 60 s and a relative mass window of 10 ppm.

After proteomic discrepancies were analyzed between the two groups, the 14 most up-regulated or down-regulated proteins in the non-invasive group were record. Among them, we chose most likely BI-related protein to stain immunohistochemically.

### Immunohistochemical staining

Immunohistochemistry (IHC) studies were performed in formalin-fixed, paraffin-embedded tissues. Consecutive 3-µm-thick sections were cut from the recipient blocks and transferred to poly-L-lysine-coated slides for IHC analysis. A modification of heat-induced epitope retrieval, involving pre-heating of EnVision FLEX Target Retrieval low pH solution to 65 °C and incubating slide for 20 min at 97 °C, followed by natural cooling to 65 °C, was used to detect the invasion-related proteins. Endogenous peroxidase activity was blocked by incubating in methanol with 0.3% H_2_O_2_ for 20 min. The sections were blocked for 60 min with 5% normal goat serum and subsequently incubated with primary antibody against the invasion-related proteins at 4 °C overnight. The antibodies and their working dilutions were as follows: Ki-67 antibody (ab15580, 1:200; Abcam Cambridge, MA, USA), glial fibrillary acidic protein (GFAP) antibody (ab7260, 1:1000; Abcam Cambridge, MA, USA) and invasion-related proteins (identified as canstatin after LC-MS/MS) antibody (ab125208, 1:1000; Abcam Cambridge, MA, USA). After washed with Tris-buffered saline, the sections were incubated in biotinylated link (Dako) for 60 min. Next, the sections were incubated in streptavidin-HRP (Dako) for 30 min at room temperature and then expression of the invasion-related proteins was visualized by a liquid DAB + substrate chromogen system (Dako).

All the staining results were positive, and were assessed by three independent pathologists, by considering the staining color value and average positive staining area percentage (APSAP). The results are divided into three levels: 1 for weak staining, 2 for moderate staining, and 3 for strong staining.

### Data analysis

All raw files were processed using the MaxQuant software (version 1.3.0.5). The generated peak list files were searched against the UniProt protein sequence database (released 2013.08 https://www.uniprot.org/). The search parameters were set as follows: enzyme selected was trypsin, with up to two missed cleavages, carbamidomethyl cysteine as a fixed modification, and methionine oxidation and protein N-terminal acetylation as variable modifications. The MS tolerance was 6 ppm, while the MS/MS tolerance was 0.5 Da. The required false discovery rate was set to 1% at the peptide and protein levels, and the minimum required peptide length was seven amino acids. At least one unique or razor peptide per protein group was required for protein identification.

For IHC statistical analysis: A chi-square test (SSPS, version 11.0; SPSS, Inc., Chicago, IL, USA) was used to determine the significance of the association between the two groups. Differences were considered statistically significant at P < 0.05.

## Results

**Fig. 1:**
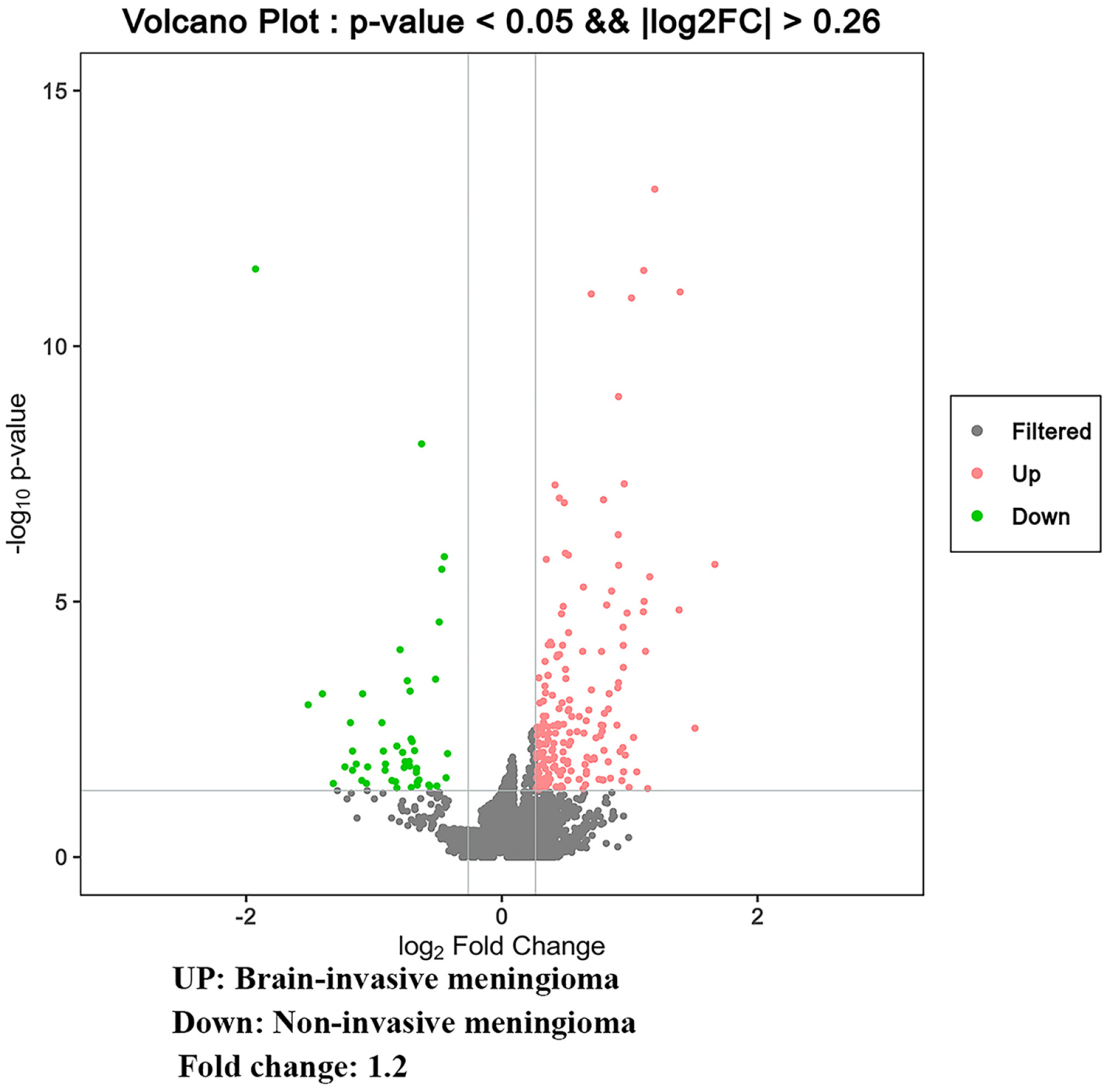
Proteomic differences between non-invasive and brain-invasive meningiomas. Volcano plot with colored, significantly-different proteins and highlighted up-regulated or down-regulated invasion-related proteins in the both groups

A total of 6498 unique proteins were identified (Supplement 1). Proteomic differences were observed between the two groups (Fig. 1). The 14 most up-regulated proteins in the non-invasive group were P10915, P03973, Q01469, O95050, P15259, Q4V9L6, P04733, Q30134, P02745, P08572, Q8IZR5, P08473, P28906 and E7EX88(Table 1). The 14 most down-regulated proteins were P17600, P14136, Q9H0Q3, Q9UQM7-2, P69905, Q16352, 95741-2, P62760, Q05315, Q13268-2, P07197, P13746-2, P02686 and Q92686 (Table 2). Among these proteins, we found that canstatin (P08572) probably related to BI and then performed immunohistochemistry study on it.

**Fig. 2:**
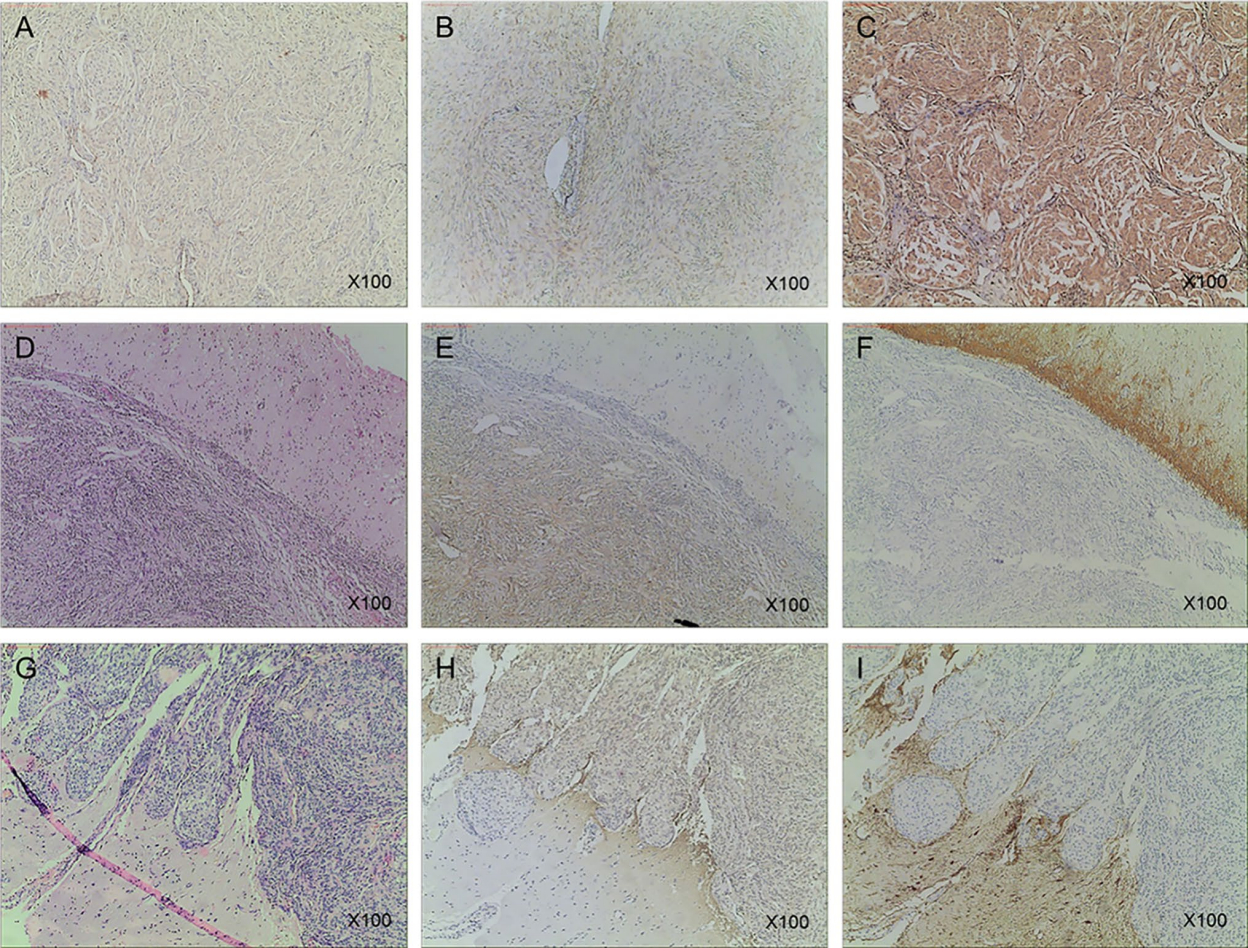
Immunohistochemical study for immune intensity of brain-tumor interface and representative staining for non-invasive and brain-invasive meningiomas. Immunohistochemical results are analyzed by determining the staining color value and average positive staining area percentage. All staining results are positive. Results are divided into three levels: 1 for weak staining (**A**), 2 for moderate staining (**B**), and 3 for strong staining (**C**). **D**, **E**, and **F** Non-invasive group (HE; canstatin, and GFAP), which shows strong staining for canstatin in the tumor mass (**E**). **G**, **H**, and **I** Brain-invasive group (HE; canstatin, and GFAP), which shows moderate intensity in tumor mass (**H**)

**Fig. 3:**
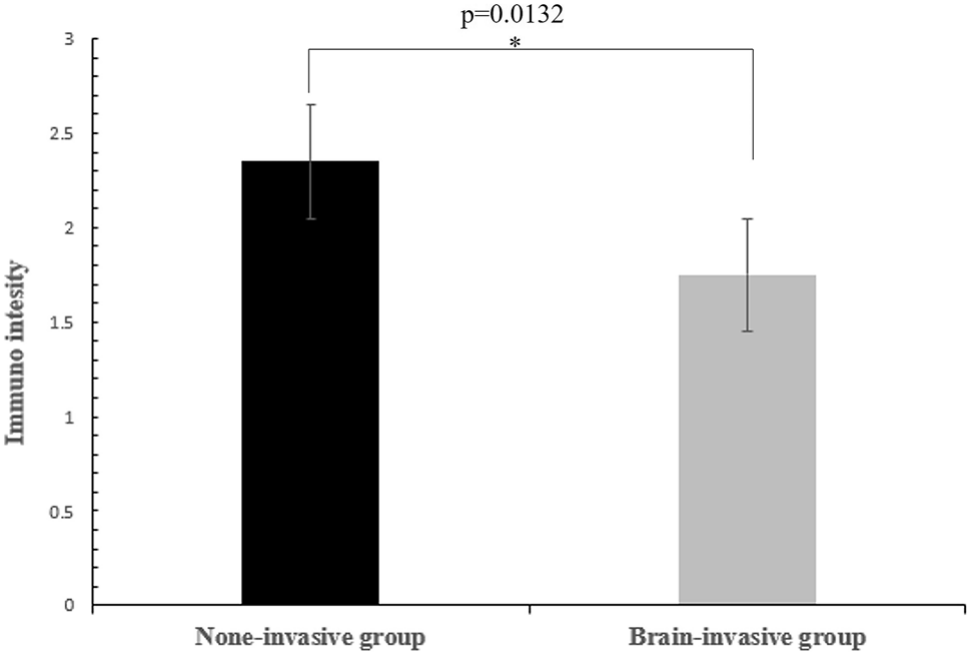
Immunohistochemical staining score of canstatin expression in non-invasive and brain-invasive meningiomas

The immunohistochemical staining results of these portions showed canstatin expression in the both groups. All the meningiomas showed positive expression of canstatin, and in non-invasive group (Avarage ± STDEV; 2.35 ± 0.74) it showed strong staining for canstatin (*p* = 0.0132) (Figs. 2 and 3) compared to brain-invasive group (Avarage ± STDEV; 1.75 ± 0.72), which showed moderate intensity in the tumor mass.

The average of Ki-67 expression was 27 ± 6.13% in the brain-invasive group, and 2.32 ± 0.56% in the none-invasive group of meningiomas.

**Table 1:** Fourteen most upregulated proteins in the non-invasive group of meningiomas

Description	Accession	Fold change
Hyaluronan and proteoglycan link protein-1	P10915	3.173
Antileukoproteinase	P03973	2.849
Fatty acid-binding protein	Q01469	2.629
Indolethylamine N-methyltransferase	O95050	2.614
Phosphoglycerate mutase-2	P15259	2.291
Transmembrane protein-119	Q4V9L6	2.229
Metallothionein-1 F	P04733	2.205
HLA class II histocompatibility antigen	Q30134	2.179
Complement C1q subcomponent subunit A	P02745	2.162
Collagen alpha-2(IV) chain (canstatin)	P08572	2.158
CKLF-like MARVEL trans membrane domain-containing protein 4	Q8IZR5	2.154
Neprilysin	P08473	2.078
Hematopoietic progenitor cell antigen	P28906	2.042
Aggrecan core protein	E7EX88	2.019

**Table 2:** Fourteen most down-regulated proteins in the non-invasive group of meningiomas

Description	Accession	Fold change
Synapsin-1	P17600	0.468
Glial fibrillary acidic protein	P14136	0.456
FXYD domain-containing ion transport regulator 6	Q9H0Q3	0.454
Isoform B of Calcium/calmodulin-dependent protein kinase type II	Q9UQM7-2	0.445
Hemoglobin subunit alpha	P69905	0.445
Alpha-internexin	Q16352	0.442
Isoform 2 of Copine-6	O95741-2	0.44
Visinin-like protein 1	P62760	0.432
Galectin-10	Q05315	0.427
Isoform 2 of Dehydrogenase/reductase SDR family member 2	Q13268-2	0.41
Mitochondrial	P07197	0.401
Neurofilament medium polypeptide O	P13746-2	0.378
Isoform 2 of HLA class I histocompatibility antigen, Myelin basic protein	P02686	0.35
Neurogranin	Q92686	0.263

## Disccusion

This study found the low expression of canstatin in meningiomas with BI. It provided a basis for understanding the molecular mechanism of BI of meningiomas and may contribute to establish molecular pathological diagnosis and identify novel therapeutic targets.

### Standard for BI in meningiomas

Systematic and accurate detection standard for BI in meningiomas contains pre-, intra-, and post-operative methods. That is imaging, intraoperative and histopathological assessment [2, 11]. BI by meningioma is defined as tongue-like protrusions of tumor cells into underlying GFAP-positive cortical parenchyma, without intervening leptomeningeal layer at the tumor-CNS interface [12]. Although at present, histopathological examination is the only standard for diagnosing BI, there is no standardized way of surgical sampling to ensure the accuracy of the detection of BI in neuropathological analysis and no clear-cut criteria exist for the histopathological detection of BI. Many studies have investigated the correlation between preoperative radiological features and BI [13–16], such as peritumoral oedema, heterogeneous contrast enhancement, and irregular tumor shape, disruption of arachnoid at the brain tumor interface, enlarged pial feeding arteries, which have been identified as predictors of BI [13–16]. In particular, edema volume was significantly and statistically related to brain-invasive meningioma [15]. However, duo to lack of definite criteria of histopathological detection of BI as a reference, there is no established radiographic criteria that clearly depicts BI. The additional intraoperative assessment regarding BI by neurosurgeon might be of clinical value for a more precise assessment of this tumor characteristic, especially in cases of incomplete sampling. But, even though under high magnification the breaching of the intervening leptomeningeal surface by meningioma tissue is detectable, it is impossible to see whether meningioma cells protrude into brain tissue. On the other hand, the intraoperative assessment is clearly prone to interobserver variance depending on the surgeons’ experience. In this study, we adopt a stricter criterion including preoperative imaging, intraoperative and histopathological assessment in order to avoid minimal histopathological criteria for calling BI in questionable samples, optimize interobserver reproducibility and ensure every invasive sample should be irrefutable before assigning.

### The role of BI in prognosis and grading

BI in meningioma is not only associated with surgical decision-making but is also independently associated with recurrence and poor prognosis [2]. But the precise definition and the prognostic role of BI remains highly controversial [5, 6, 8]. But even so, BI in meningioma was clearly defined as an additional criterion for atypia in the revised fourth edition of the 2016 WHO classification of CNS tumors [17]. The 2021 WHO criteria kept BI as a standalone diagnostic feature for grade 2 meningiomas [18]. Because the majority of brain-invasive meningiomas also show other high-grade (atypical) features (such as elevated mitotic activity) and BI in previous grade II and III tumors is no longer disputed, only a subset of BI otherwise benign meningiomas, that is meningiomas classified as grade 2 solely on the presence of BI, remains controversial. Since many changes occurred in the WHO criteria in this decades, it must confound interpretation of meningioma grading in this study (2005–2021). That is earlier grade I meningiomas likely contained many grade II samples in this study. In order to eliminate this confusion of grading, we only collect invasive cases in WHO grade III meningiomas met invasive criterion and collect non-invasive cases in non-invasive grade I meningiomas to exclude WHO grade II meningiomas which may include a subset of BI otherwise benign meningiomas with controversial.

However, inconstant assessment of BI may be a major key point causing previous debates about the prognosis value of BI [2, 6]. Intraoperative tumor sampling is non-standardized and especially areas of interest may not always be amenable to appropriate sampling [11].

There are several factors that can potentially influence the intraoperative sampling. In cases of skull base meningiomas, the trend of performing smaller and more focused surgical approaches will very likely contradict optimal intraoperative conditions for sampling. When meningiomas is adjacent to highly eloquent areas, especially if adhesions or possibly infiltrative growth is present, it is most important for neurosurgeons to leave the arachnoid membrane intact to avoid damaging cortex and do not expand sampling to include bordering cortical tissue [19]. If no brain tissue is detectable, evaluation of BI is not possible. In neurosurgical practice, meningiomas are usually resected piece-meal by suction and Cavitron Ultrasonic Surgical Aspirator (CUSA). The use of CUSAs, with subsequent tissue loss may further lead to the difficulty in selective sampling of the interface in meningioma tissue and no dedicated specimen from the tumor surface for neuropathological analysis was obtained [20]. It is documented that the more specimens available, the more BI observed [21].

On the other hand, the histopathological characteristics used to determine BI are not clearly defined [2]and possibly vary between departments and neurooncological centers. Further, variations in interobserver interpretation and different staining protocols make it difficult to establish clear cut off values.

### Molecular mechanisms of brain-invasive and LC-MS/MS-based proteomic

Since BI and grading of meningiomas is based on subjective assessment of histopathological findings, this system is suboptimal with problematic interobserver variation. Advances in molecular characterization of meningiomas have revealed several genetic aberrations and driver mutations. Molecular classification of meningiomas based on copy number variation, point mutations, methylation, and transcriptomic and proteomic data stands out as a future diagnostic work-up of meningiomas [22]. Thus, WHO CNS 5th endorses molecular biomarkers to refine classification and malignancy grading. Some studies have focused on molecular mechanism and searching for molecular biomarker expression that could correlate with BI, in order to avoid subjective assessment of histopathological findings with problems of interobserver variation.

It has been documented that BI in meningioma is correlated to molecular alterations at various cellular components and in signal transmission pathways [23]. Such alterations result in three-step process, initially degradation of ECM/BM, and tumor cells migration, finally promoting adhesion of meningioma cells to resident cells with the help of growth factors and blood-vessel formation [24], leading to the tendency of the tumor to infiltrate and local BI.

Since BI related molecular alterations depend on protein dynamics, demonstrating changes from the proteomic perspective enables us to understand mechanism of the BI. From body fluids analysis, proteomics could identify possible biomarkers of BI, such as those proteins secreted by pathological cells or affected by BI processes. Liquid chromatography-tandem mass spectrometry (LC-MS/MS) is the standard laboratory technique for the analysis of biological fluids. It has reached maturity so that most small-molecule concentrations in the lower picomolar range can be successfully assessed. In this study, LC-MS/MS-based proteomics showed that canstatin was down-regulated in the brain-invasive group, which indicated that canstatin may contribute to the inhibition of BI.

### The role of canstatin in inhibition of BI

In fact, canstatin is closely related to ECM/BM-one of the components of BI. Basement membrane is mainly composed of type IV collagen which has been recently identified being involved in the regulation of tumor angiogenesis [25]. Type IV collagen has six different α-chains, α1–α6. The triple helix of type IV collagen consisted of two α1-chains and one α2-chain is ubiquitously expressed in the basement membrane of whole body. C-terminal domain of type IV collagen called non-collagenous 1 (NC1) domain plays an important role in the assembly of α-chains [26]. During tumor progression and metastasis, the type IV collagen is degraded by ECM-degrading enzymes, such as matrix metalloproteinase (MMP)-2 and MMP-9 [25].

Canstatin is a 24 kDa non-collagenous C-terminal fragment cleaved from type IV collagen α2-chain. It was firstly identified as a recombinant protein with potent anti-angiogenic and anti-tumor activities [27]. Canstatin inhibits angiogenesis through the inhibition of proliferation, migration and tube formation in vascular endothelial cells [27], through the regulation of the Akt and FAK pathways [28]. Canstatin also induces apoptosis in vascular endothelial cells [27], by inhibiting the FAK/Akt pathway in vascular endothelial cells [29], or by activating of the Fas-dependent apoptotic pathway [28].

Currently, endogenous angiogenesis inhibitors, which are mainly proteins or fragments formed in vivo, are widely used due to their non-toxicity, lower drug resistance, high tolerance [30], particularly endogenous angiogenesis basement membrane collagen-derived inhibitors [31]. Since canstatin originates from endogenous type IV collagen α2-chain expressed in the whole body, it may have fewer side effects than the approved chemotherapeutic agents. It suggests that canstatin is not only an attractive molecular biomarker but also probably be a novel therapeutic target for BI of meningiomas.

## Conclusion

Our results demonstrated the low expression of canstatin in brain-invasive meningiomas, a finding that may contribute to the development of new molecular diagnosis and therapeutic tools for the BI of meningiomas.

## Supplementary Information

Below is the link to the electronic supplementary material.

Supplementary material 1 (XLSX 533.3 kb)
